# The “Too-Few Elephant Problem”: Quantitative Analysis of Historical Elephant Tree-Toppling Rates Across Africa

**DOI:** 10.1007/s10021-026-01089-5

**Published:** 2026-08-20

**Authors:** Sally Archibald, Anya P. Courtenay, Fezile Mtsetfwa, Domingos Fortunato P. F. da Silva, Katherine Bunney, Robert Guldemond, Penny Mograbi, Caitlin Ransom, Casey M. Ryan, Norman Owen-Smith

**Affiliations:** 1 School of Animal, Plant and Environmental Sciences, University of the Witwatersrand, Johannesburg, South Africa; 2 School of GeoSciences, University of Edinburgh, Edinburgh, UK; 3 Royal Botanic Garden Edinburgh, Edinburgh, UK; 4 Utrecht University, Utrecht, Netherlands; 5 Department of Exact and Natural Sciences of ISCED-Huambo, Avenida Allioun Blondy Beye - Bairro Académico, P.O. Box No. 2376, Huambo, Angola; 6 Elephants Alive, P.O. Box 960, Hoedspruit, 1380 South Africa; 7 Conservation Ecology Research Unit, Department of Zoology and Entomology, University of Pretoria, Pretoria, South Africa; 8 South African Environmental Observation Network, Pretoria, South Africa

**Keywords:** Elephant Impacts, Tree Loss, Above-Ground Biomass, Megaherbivore Restoration, Elephant Density, Rewilding, Tree Topkill, Vegetation Dynamics, Degradation

## Abstract

Elephants were a major driver of tree stem mortality in Africa, but their impacts are now spatially constrained and they no longer topple trees across the continent. Thus, most African landscapes lack a key ecological agent that helped maintain diversity and functioning. Even ecologists living in Africa fail to anticipate the rampant destruction of trees that can occur when elephants live in and move through landscapes. If we can build in a consensus on the degree of above-ground tree turnover that is inherent in different contexts, it would inform restoration projects and ecological definitions of degradation. We collated empirical data on treefall in ecosystems with and without elephants and predicted past tree loss spatially. The metrics used (%trees or #trees ha^−1^) reveal assumptions about the processes controlling elephant damage. On average elephants knock down 5.4 [IQR 3.0–8.3] % of the trees in an ecosystem per annum. Mapping this into places that no longer contain elephants reveals the above-ground biomass turnover that would have been the norm for millennia before elephants were widely exterminated. On average, this is ~ 1/3 of woody biomass production but at a local scale it can often exceed production. There is an urgent need for better theory on elephant tree interactions, and restoration projects should embrace the rapid turnover dynamics these ecosystems evolved with.

**Supplementary Information:**

The online version contains supplementary material available at 10.1007/s10021-026-01089-5.

## Highlights

- Elephants, where they are still present, are responsible for toppling between 3 and 8% of the trees in African savanna ecosystems annually.
- Treefall rates in ecosystems with elephants can exceed regeneration/recruitment rates, driving a dynamic vegetation layer that shows high spatial and temporal variability.
- Restoration projects should embrace the rapid turnover dynamics that these ecosystems evolved with.

## Introduction

The “elephant problem” has long troubled ecologists. Ecosystems with elephants are often exposed to rates of above-ground tree biomass turnover that appear unsustainable (i.e. tree loss exceeds tree recruitment). The result can be open, grassy or shrubby savannas maintained through top-down control by elephants (Buechner and Dawkins 1961; Dublin and others 1990; O’Connor and others 2024), that appear degraded by some definitions, and may have knock-on effects on other aspects of biodiversity (Kerley and others 2008; but see also Guldemond and others 2017).

Managers of almost all protected areas in Africa containing elephants have, at one point or another, had to face the question of whether they should reduce their elephant populations to safeguard their tree populations. The justification for intervening has been that elephants in the past were free-ranging and did not stay long in any one landscape, and that their impacts have been exacerbated by land use change and fences blocking wider movements. Elephant impacts in conservation areas may therefore not be representative of their past impacts, and there is consequently a need for active management of elephant populations (as discussed by Laws 1970; Anderson and Walker 1974; Guy 1976; and more recently by O’Connor 2017). However, this idea has been challenged by researchers who view even very high elephant impacts as fundamental to ecosystem functioning, and envisage African ecosystems (and other ecosystems that used to contain megaherbivores) as intrinsically dynamic with large variations in tree cover (Caughley 1976), or periodic switching to alternative ecosystem states (Dublin and others 1990), enabled by what others would call unsustainable impacts (Lewis 1991; Scholes and Mennell 2008; Guldemond and others 2017).

This debate highlights that we know very little about the spatio-temporal dynamics of elephants when they were free roaming through Africa. Early European explorers recorded elephants in regions where they have long since vanished, and in numbers far larger than those seen today. William Cotton Oswell encountered “a herd of at least four hundred elephants” in the Kalahari north of the Molopo River, on today’s South Africa–Botswana border, in 1849 (Oswell 1900). David Livingstone also reported “prodigious numbers” of elephants in the middle Zambezi valley of south-central Africa, including one herd estimated at over 800 individuals (Livingstone 1875). In northern Namibia, Charles Andersson observed elephants “as thick as cattle” around Lake Ngami at the edge of the Kalahari Desert, and reported that whole villages were sometimes overrun at night by passing herds (Andersson 1857). Together, these accounts highlight the abundance and breadth of elephant distribution in the 19th century. This is corroborated by current-day observations: regional densities in landscapes where they still move with relative freedom can easily exceed three elephants per km^2^ (Gibson and others 1998; Tinley 1977) and analyses of landscape use from radio-collar data identify almost all of sub-Saharan Africa as suitable elephant habitat (Wall and others 2021).

Nineteenth century observers also remarked on vegetation impacts by elephants, including toppling mopane (*Colophospermum mopane*) and “levelling the forest” in parts of Southern Africa (Selous 1881; Gordon-Cumming 1855). These records attest to the persistent role of elephants in toppling trees and structuring savannas. Subsequently, a trend towards thickening of the woody plant cover has been documented in many ecosystems, except where elephants remain (Stevens and others 2016).

By the early 19th century, elephants had already disappeared from much of the Cape and North Africa (Carruthers 1995). The expanding ivory trade accelerated these losses and at its height in the late 19th century between 8000 and 30,000 tusks were shipped annually from Zanzibar (Coutu and others 2016; Beachey 1967), and ivory trade from French West Africa averaged over 10,000 Mg a year throughout the 19th and well into the 20th century (Roth and Douglas-Hamilton 1991). By the 1880s, elephants were scarce in East Africa’s coastal hinterlands, while in Southern Africa, most of the large populations were eliminated between 1800 and 1870 (Bunney 2014). This contraction marked a profound shift in the historical geography of elephants, as animals once spread across the continent were reduced to fragmented strongholds in just a few decades. When elephant numbers started to climb in some parts of Africa in the 1960s, having dramatic impacts on the vegetation (Spinage 1973), researchers began to problematise elephants and constructed the “elephant problem”, without any first-hand knowledge of what ecosystems with ecologically relevant densities of elephants in them should look like.

The intense concern with the “elephant problem” has provided us with an extensive and expansive literature on elephants and their impacts, which could be harnessed to paint a picture of how these ecosystems might have looked when elephants were ranging freely through them in numbers that we struggle currently to imagine. So, turning this issue on its head, this paper aims to address the “lack-of-elephant problem”. How exactly have our ecosystems without elephants been affected by their loss, and what levels of tree turnover should we be aiming to reintroduce into these systems in order to restore their functioning (Søndergaard and others 2025)?

### Predicting Elephant Impacts: Drivers of Tree Toppling Rates

The literature on elephants and their impacts is vast, yet difficult to generalise (Guldemond and Van Aarde 2008). Data are often collected to address a specific local research or management challenge, e.g. (Guy 1976), using a wide range of metrics that are not always comparable. There is also the likelihood that elephant damage is only studied/reported in contexts where elephants are noticeably having an impact i.e. available studies overplay their overall ecosystem effects. Despite these challenges, elephants are known to show predictable patterns of habitat use within landscapes and to have strong selectivity for particular tree species and tree size classes. Elephants, particularly bull elephants, spend the majority of their time close to water (within 5–10 km) (Skarpe and others 2014; MacFadyen and others 2019), at lower elevations (Asner and others 2015; Mograbi and others 2017), and on flatter slopes (Teixeira and others 2025). These alluvial habitats often have higher clay contents and soil nutrients (Skarpe and others 2014), and some studies suggest that elephants are more likely to damage trees on clay, basalt, and gabbro soils (Eckhardt and others 2000; Shannon and others 2008). Within landscapes, therefore, there are predictable high and low zones of elephant impact.

Elephant tree top-kill rates are known to be highest in the intermediate tree size classes (6–40 cm diameter at breast height (DBH), or 3–9 m tall (reviewed by Mtsetfwa and Archibald (2026), Table S2, Figure S5). Here, we refer to “topkill” as the death of a tree’s above-ground stems and canopy with the unknown potential for below-ground roots and basal buds to remain alive and capable of resprouting. Trees larger than 40 cm in diameter are resistant to being felled, unless subject to trunk core rot, but can still be damaged by bark stripping, which can increase the risk of top-kill through other processes (fire, disease, and termites) (Helm and others 2011). Thus, heavily impacted elephant areas tend to consist of “browsing lawns” of resprouting saplings at the preferred height and diameter for elephant browsing (O’Connor and others 2024; Tripathi and others 2019). Moreover, elephants also show strong selection for or against particular tree species and may topple almost all of one preferred tree type before impacting any other species (Cook and others 2017; Harrington and Ross 1974; Thomson 1975).

Several studies show that higher elephant densities result in higher top-kill rates, especially when accounting for differential impacts of bull elephants (Jachmann and Croes 1991; Shannon and others 2008; Asner and others 2015). However, other studies have found little connection between the density of elephants and their impacts on trees (Guldemond and Van Aarde 2008; O’Connor and others 2024; Ferry and others 2021). For example, Smallie and O’Connor (2000) found that elephants foraged largely on previously hedged mopane in Venetia Game Reserve, and the high availability of this forage reduced the top-kill of larger trees. Moreover, elephant effects on tree populations can be highly stochastic, driven by environmental factors rather than elephant density: Chafota and Owen-Smith (2009) report several instances where up to 80% of the trees in a population were toppled after frost, fire, or drought removed all other available elephant forage.

### Tree Density Impacts on Elephant Toppling Rates

One particularly underexplored determinant of tree toppling rates is the density of trees in a landscape, which is key to predicting the trajectory of elephant impacts over time, and for generalising across different landscapes. The literature on this is perplexing. Modelling studies tend to use absolute loss rates (trees toppled per elephant) and assume that damage is independent of tree density (Barnes and others 1994; Baxter and Getz 2005). This is supported by some field observations (Lewis 1991), but Dunham (1989), Hema and others (2017) and Tafangenyasha (1997) all show tree toppling rates to increase with tree density, implying that *relative loss rates* are more appropriate metrics to use. In a large study across a range of landscapes in Uganda, Wing and Buss (1970) concluded “Considering the vast difference in total acreage and numbers of stems per acre in each cover type there is a surprisingly small amount of variation in the average percentage of stems utilised… this relatively constant utilisation percentage, however, produces a greatly different number of total utilised stems.”

Clearly the metric used to describe the elephant impacts implies strong assumptions about how elephant damage interacts with tree presence and this does not seem to have been satisfactorily addressed in the literature, although it would be important for generalising elephant impacts across ecosystems with very different tree densities.

### Rationale

Currently, our ability to provide answers to simple questions about levels of elephant impacts that are ecologically appropriate remains limited (Scholes and Mennell 2008; Guldemond and others 2017). Existing quantitative meta-analyses have assessed whether elephant impacts are positive or negative, rather than trying to predict rates of change in tree populations. To fill this gap, here, we collate and compare quantitative information on elephant tree-toppling rates from 73 field studies in savanna and woodland ecosystems in Africa to provide estimates of the amount of tree turnover that could be expected in different vegetation types and for varying elephant densities, and to extrapolate this across regions currently lacking elephants. We also aim to mobilise researchers to expand beyond localised field studies to address some of the theoretical and empirical challenges that prevent us from understanding elephant impacts at larger scales.

## Methods

### Literature Search

We used the systematic reviews performed by Mtsetfwa and Archibald (2026, 50 papers), and Guldemond and others (2017, 73 papers) and identified all papers that report quantitative measures of tree toppling (or top-kill) rates for savanna elephants (*Loxodonta africana*). We sourced additional papers from references and citations within these papers, and from a database on elephant impacts maintained by Norman Owen Smith.

We included papers where the elephant impacts were reported over a set time period, and a unit area so that we could extract either absolute rates (number of trees toppled per ha per year) or relative rates (% trees toppled per year). We used year as a minimum unit of measure as elephant impacts vary by season—increasing in the dry season when elephants are largely browsers, and also varying in areas where elephants migrate seasonally in and out of landscapes. To be included in our database, the data had to encompass at least one dry season period. In cases where the impacts were reported over longer time periods (e.g. biennial for Asner and others (2015) and Mograbi and others (2017), or triennial for Shannon and others (2011)), we divided by the number of dry seasons to get an annual rate. Where possible we extracted information on elephant densities (elephants km^−2^) to link with the treefall rates. When background rates of treefall were available (either due to exclosures or contrasting land use studies), this was also recorded. Sometimes the authors only considered trees in particular size classes or impacts on specific tree species. We noted the size class information but excluded any studies that focused on particular species, as not being representative of landscape-level rates.

Elephant impacts are stochastic in space and time (Chafota and Owen-Smith 2009). To be included in the analysis, studies had to be at a scale large enough to represent landscape-level, rather than plot-level impacts (i.e. integrating across spatial variation associated with catenal position and distance to water). Where field data were reported for individual monitoring plots, we averaged these to produce a landscape-level value. This analysis therefore differs from most previous modelling studies that investigated the drivers of within-landscape variation in elephant impacts (Shannon and others 2008; Asner and others 2015; Mograbi and others 2017).

Additional information on elephant densities, the soil and vegetation type, the size of the study area, and the type of data collection was collated. Sites were classified from information provided in the papers into one of five vegetation formations (Osborne and others 2018): mixed Acacia woodland, mixed Combretum woodland, Detarioideae (Miombo/Sudanian) woodland, Mopane woodland, and grassland-forest mosaics. We had no a priori expectations about which vegetation types would be most affected, but expected that differences in tree architecture and in elephant preference for the dominant species would cause differential impacts (Teren and others 2018). Methods used in papers ranged from repeat field sampling, to aerial photography, to aerial lidar height change detection. Field data were often reported by size class and we summed values across all size classes to obtain landscape-level tree toppling rates for comparison with aerial studies. The final dataset comprised 73 records scattered across eight countries in Africa that reported change over time at scales large enough to be included in a landscape-level meta-analysis.

Throughout this manuscript, we refer to “top-kill” or “stem toppling”, the death of a tree’s above-ground stems and canopy with the unknown potential for belowground roots and basal buds to remain alive. Actual stem mortality from elephant impacts is rare as most individuals resprout vigorously when their stems are damaged. Aerial methods reviewed in this paper would not be able to assess stem mortality. Furthermore, field-based observations of top-kill versus mortality are difficult to determine without monitoring a stem over multiple censuses as stems presumed to be top-killed in one census are frequently found as resprouting in a subsequent census. However, when combined with intense fires, stem mortality can occur and tree population size as well as structure can be affected. Whether stems are top-killed or killed will affect the recovery rates after elephant impacts, and needs to be further quantified.

### Data Collation and Cleaning

We analysed records in terms of the absolute treefall rate (trees toppled per ha per year) as well as the relative treefall rate (% trees toppled per year). Where the total number of trees in a landscape was reported, it was possible to convert between these two metrics. These were quantified for:

- landscapes with elephants (i.e. including elephant damage as well as background rates of top-kill caused by senescence, cyclones, fire, and disease)
- background rates in landscapes where elephants were excluded landscapes where elephants were excluded (experimental exclosures and land use comparisons),
- studies where only elephant impacts were recorded by distinguishing elephants from other forms of damage in the field.

We subtracted the mean background rate over all studies that reported background rates to extract a metric of the treefall caused by elephants alone for each study site.

1 $$ {\mathrm{LR}}_{{{\rm{ele}}}} = {\text{ LR}}_{{{\rm{ls}}}} - {\mathrm{mean}}\left( {{\rm{LR}}_{{{\mathrm{bg}}}} } \right) $$

where LR_ele_ is the elephant loss rate for that site, LR_ls_ is the landscape-level loss rate, including all other drivers of top-kill, and LR_bg_ is the background rate of tree turnover in ecosystems without elephants. See Figure S4 for tests of assumptions around additive effects of elephant on background rates.

### Analysis

We chose to do all further analysis on relative loss rates (% toppling), partly because this was the more commonly reported metric (larger sample size) and partly because it is more reasonable when extrapolating to other circumstances (to avoid estimating higher top-kill rates than there are trees present for example, but see discussion). We used generalised linear regression models to test whether elephant densities or vegetation type could explain any variance in tree toppling rates. The response variable was the proportion of trees toppled (positive, left-skewed) so we used a gamma distribution with a log link (values never got close to zero so beta-regression not necessary). We used these models to produce estimates of the mean and standard deviation of treefall rates that we could use to extrapolate past elephant impacts across different landscapes in Africa. To find the model with the best explanatory power, we compared model performance using the Performance package in R statistical software.

### Converting Treefall Rates to Biomass Removed

To convert tree toppling rates into estimates of biomass removed, we needed to incorporate information on the size classes of trees likely to be toppled and their above-ground biomass. This was summarised by Mtsetfwa and Archibald (2026): ~ 65% of toppled trees are between 6 and 40 cm diameter at breast height (DBH) (3-9 m height), ~ 15% between 0 and 6 cm DBH (0-3 m height), ~ 20% are > 40 DBH (> 9 m height) (Table S2). To convert to above-ground biomass, we used field plot data from 391 plots across a rainfall gradient in Southern Africa (representing 41,452 individual stem records) and calculated the distribution of biomass in these three size classes (0–6 cm, 6–40 cm, and > 40 cm) (Table S2).

Using a map of above-ground biomass (AGB) for Africa (Bouvet and others 2018) aggregated to 1 km resolution, we allocated AGB to the three tree size classes based on their relative contribution to plot-level AGB in the SEOSAW plots. We then removed biomass from each size class in proportion to the elephant preference (Table S2) and summed these values across all size classes to produce an estimate of the total biomass removed per 1 km pixel (deltaAGB). This equation does not include an effect of elephant density, which had low explanatory power in our model (see below).

2 $$ \delta {\text{AGB }} = {\text{ sum}}\left( {{\text{ AGB}}*{\text{ fAGB}}_{{[{1}..{3}]}} * \, \left( {{\mathrm{LR}}_{{{\mathrm{ele}}}} } \right)*{\mathrm{fToppled}}_{{[{1}..{3}]}} } \right) $$

where AGB is a raster of estimated above-ground biomass in Mg from Bouvet (Bouvet and others 2018) radar data at 1 km resolution, fAGB_[1..3]_ is the proportion of the total biomass that is found in each size class (summing to 1, Table S1), LR_ele_ is the fraction of trees toppled by elephants per year (derived from our glm models), and fToppled_[1..3]_ represents how tree loss is distributed between the three size classes (proportion summing to 1, Table S1). δAGB therefore represents a raster of 1 km resolution of the above-ground biomass removed by elephants in Mg year^−1^ when they were free-ranging through Africa. Our understanding of the past extent of *L. africana* is limited, but it is assumed that they ranged widely across most open ecosystems, although with large seasonal and inter-annual variation (Purdon and others 2018). The scaling-up analysis was therefore performed in all African ecosystems except forests and Madagascar (which never contained elephants).

### Comparing Elephant AGB Turnover with Above-Ground Production and with AGB Turnover from Woodfuel Harvesting

To contextualise our estimates of past tree turnover rates caused by elephant, we compared these results with newly published estimates of NPP and mortality from Godlee and others (in review), as well as from a recently published spatial dataset on human woodfuel harvesting rates (MoFuSS, A Ghilardi and Bailis 2024). Using the MoFuSS dataset, we mapped places in Africa where current woodfuel harvesting is higher than our maximum estimates of past elephant toppling rates, and where it is lower than our minimum estimates of past elephant toppling rates, to assess how much of Africa is still experiencing “reference” levels of tree turnover. The MoFuSS data focuses on tree losses for woodfuel, and does not include losses due to timber production, honey harvesting, or clearing for agriculture, and is therefore not an estimate of the totality of human impacts.

## Results

**Figure 1:**
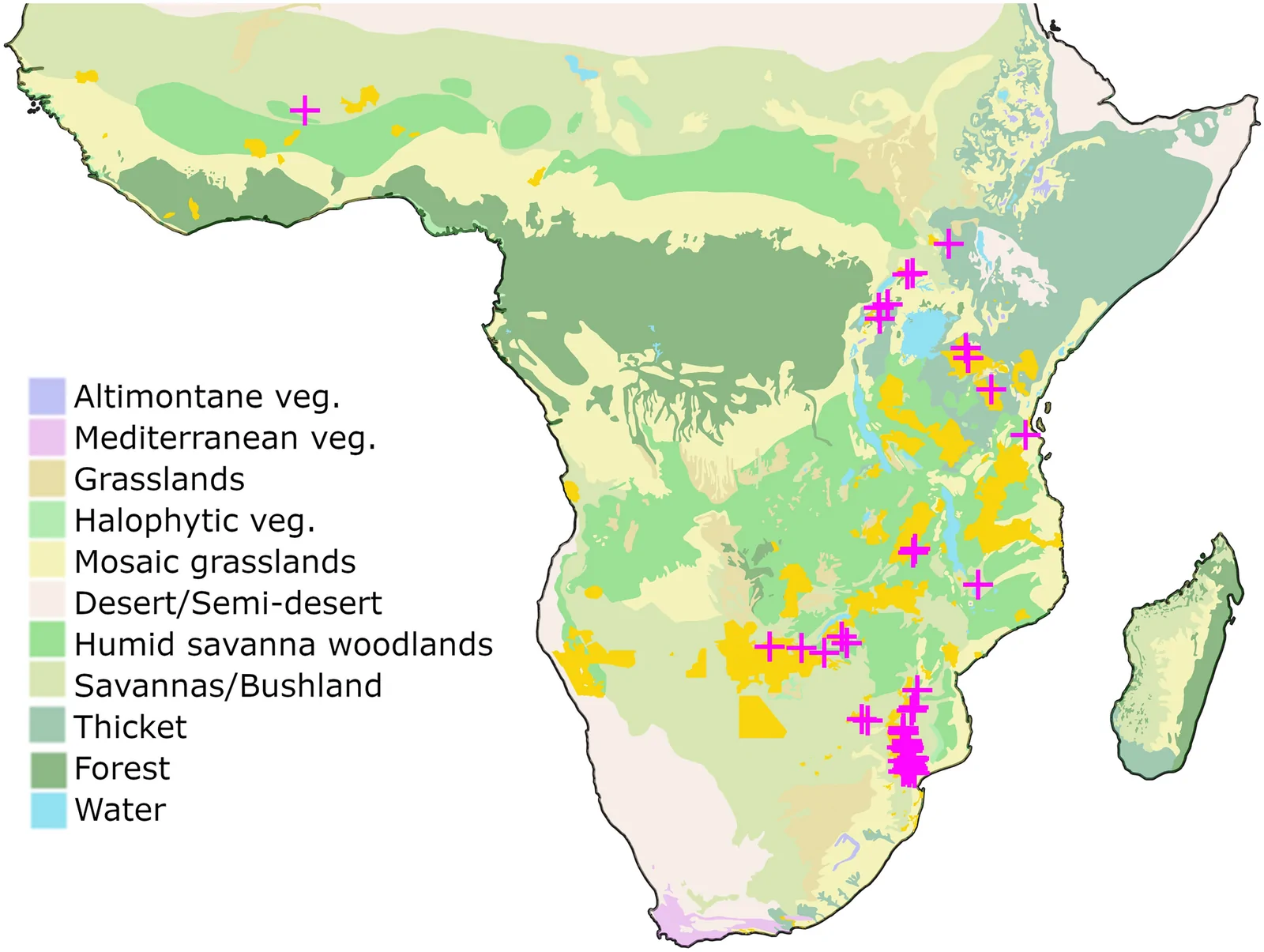
Location of the elephant toppling rates datasets used in this analysis (pink) in relation to dominant vegetation, and the existing landscapes that still contain elephants (dark yellow, from Huang and others (2024)). Vegetation map is derived from White (1986). The studies cover a wide range of ecosystems and are spread across most of the landscapes still containing elephants, but there is only one quantitative study from West Africa. Almost half (32) of the studies were from the Kruger National Park in South Africa, eight were from the Serengeti-Mara in Tanzania, and six from the Sengwa Wildlife Research Area in Zimbabwe. Elephants have never been present in Madagascar.

We found 73 studies from 22 African ecosystems that provided quantified estimates of the proportion of trees toppled by elephants (Figure 1, Table S1). These studies were highly skewed toward a few protected areas and countries (Figure 1), but did cover all major vegetation types in Africa, except forest.

**Figure 2:**
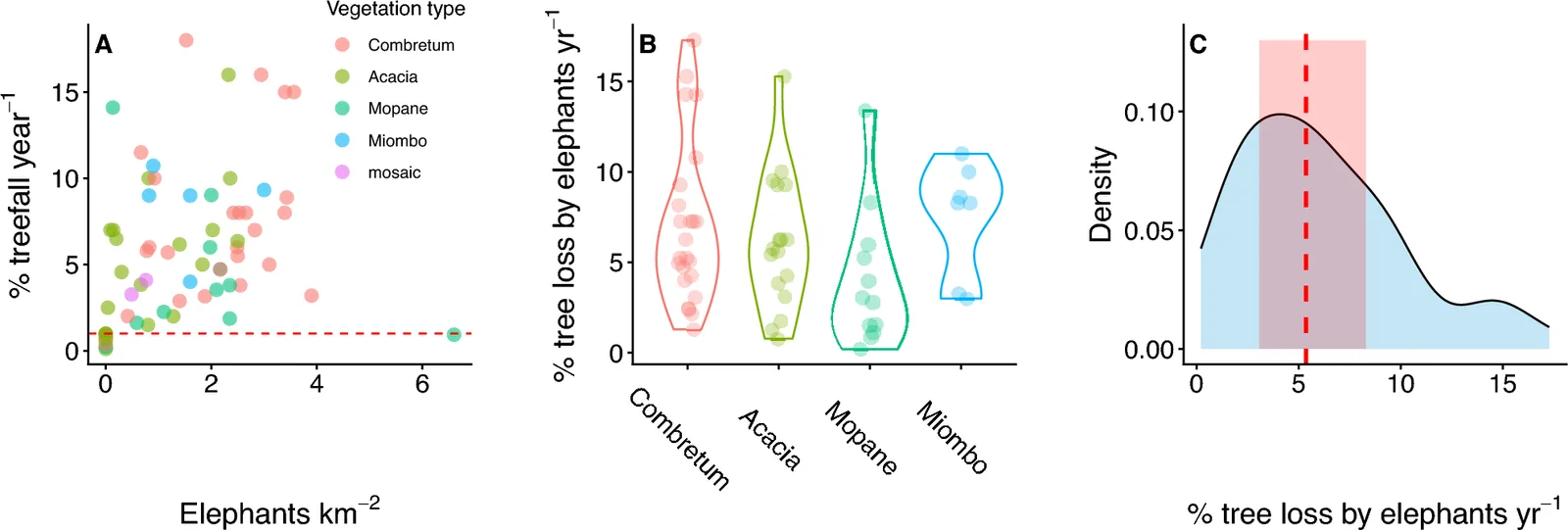
**A**. Ecosystems with elephants have ~ 10 times higher treefall rates than ecosystems without elephants (*β* = 2.33, *t*-value = 11.236, *p* < 0.001), but elephant density does not increase the explanatory power of this relationship (Table S3): Red line is the maximum background tree turnover reported without elephants (1%) and the outlier value with elephant density > 6 km^−2^ is a landscape in the Letaba area of the Kruger National Park (Asner and Levick 2012; Siebert 2010). **B** Vegetation type does not explain variation in elephant impact (*F* value = 1.151, *P* = 0.34). Forest-grassland mosaics were excluded as they were represented by only two data points. **C** Our best estimate for the elephant treefall rates in any part of Africa is the median rate (red-dash line) recorded in all studies. i.e., 5.4 [IQR 3.0–8.3]%.

Elephant-caused stem turnover ranges from 3 to 8% of the trees in a landscape (median 5.4% Figure 2, Table 1), and this rate is on average ten times higher than background rates in landscapes without elephants (*β* = 2.33, *t*-value = 11.236, *p* < 0.001). The effect of elephant presence is highly significant, but adding elephant density or vegetation type did not improve the explanatory power of the model (Figure 2A and B, Table S3). When these relative rates were extrapolated across ecosystems in Africa that used to have elephants present, and converted into above-ground woody biomass (AGB) based on size class preferences, we predict tree AGB turnover rates due to elephants of 0.59 Mg ha^−1^ (median, IQR 0.05–1.05 Mg ha^−1^ yr^−1^), increasing with mean annual rainfall (Figure S1).

**Table 1:** Quantifying Mean and Median Tree Loss Rates (% Loss per Annum) in (i) Landscapes with Elephants, (ii) Landscapes without Elephants (Background), and (iii) the Isolated Effect of Elephant Toppling (Elephant Toppling Rates). See Methods for How These Values Were Calculated from the Dataset of Which There Were Studies That Reported All Three Metrics.

	mean % tree loss	sd % tree loss	Median % tree loss	q25 % tree loss	q75 % tree loss
Landscape tree loss rates with elephants	6.86	4.03	6.08	3.80	9.02
Background tree loss rates	0.67	0.35	0.73	0.66	0.90
Elephant toppling rates	6.14	4.03	5.36	3.07	8.29

## Discussion

We showed that tree loss rates in ecosystems with elephants are on average ten times higher than background treefall rates without elephants and range from 3 to 8% of the trees in a landscape (Figure 2, Table 1). Although these tree loss rates might seem high, it includes all tree individuals toppled and elephant impacts are far higher on small to medium-sized trees, seldom toppling very large (> 40 cm) individuals. Thus, the equivalent woody biomass turnover by elephants averages 0.59 Mg ha^−1^ (median, IQR 0.05–1.05 Mg ha^−1^ yr^−1^) and increases with rainfall (Figure S1). This is comparable to the total turnover rates of 0 to > 1 Mg ha^−1^ yr^1^ reported from long-term field plots in the region (Godlee and others in review), some of which have elephant present.

Are these loss rates sustainable; i.e. how do they compare with biomass production rates in the same ecosystems? Godlee and others (in review) report wood production across tropical savannas and dry forests of 1.18 MgC ha^−1^ y^−1^ (95% CI: 0.99–1.36 MgC ha^−1^ y^−1^), which, when converted to biomass, represents ~ 2.8 times what we estimate elephants would have been removing in the same environments. However, plotting the interquartile range of elephant loss against wood production demonstrates that tree turnover from elephants frequently exceeds replacement rates at any one point in space and time (Figure S1). Thus, on average, elephants would have been knocking down about 35% of the annual wood production across African savannas and woodlands, but their short-term effects would often have been higher than replacement rates (Figure S1). This would have been in addition to the already high turnover caused by fire and other disturbances. The conclusion is that across large areas in Africa, tree top-kill was frequently equivalent to, or even higher than, tree recruitment, and we should be managing to maintain and enable these dynamics. Certainly, the trees in these ecosystems show strong adaptations to top-kill and resprout vigorously across a range of size classes (Msetfwa unpublished data), supporting the idea that they evolved in ecosystems with high disturbance impacts on trees as well as saplings.

We found highly variable impacts within vegetation types, but no evidence for higher impacts in certain ecosystems. A closer look at the literature highlights our ignorance about which ecosystems would have been more impacted by elephants in the past: Jachmann and Bell (1985) argue elephant impacts would have been highest in oligotrophic Miombo woodlands, and Skarpe and others (2014) also proposed that low-nutrient ecosystems would have higher elephant damage. In contrast, other authors have highlighted that many common Miombo species (e.g. *Baikiaea plurijuga, Ochna pulchra* and *Erythrophleum africanum)* are often avoided by elephant (Owen-Smith and others 2019) which preferentially utilise the other habitat types within this ecoregion (Ribeiro and others 2008). However, the dominant Miombo genera: Brachystegia and Julbernardia are frequently reported as being eaten, some even preferred (e.g. *Brachystegia boehmii*) (Holdo 2006; Ransom 2019), and within our dataset, there were very high utilisation levels in some Miombo sites. We can infer that there are sufficient trees attractive to elephants in any tree community, and we can infer that elephants would have been toppling trees across the continent and in all vegetation formations. However, analyses of stems alone such as this one, that do not distinguish species, can obscure the important and significant shifts in composition that can occur within vegetation types as preferred species are lost from the ecosystem to the benefit of less preferred species (Mapaure and Moe 2009; Teren and others 2018). Meta-analyses on the effects of their selective usage on species composition would be highly informative (Owen-Smith and others 2019).

The limited explanatory power of elephant density is surprising: sometimes, very high elephant densities had low reported treefall rates (e.g. 0.93% elephant toppling in Mopane shrublands in Letaba with an estimated six elephants km^−2^: Figure 2A). This is probably related to past history of elephant impacts and initial tree structure: Smallie and O’Connor (2000) describe how when elephants move into an ecosystem, they exert initially very high rates of treefall until there are sufficient branches at preferred elephant browse level, after which they preferentially browse these resprouting individuals rather than toppling further trees. Lewis (1991) showed that the amount of damage on trees depended on the amount of shrubs and coppicing vegetation—when this was higher, tree damage was lower. When ecosystems are in a “browsing hedge” state—with a majority of trees maintained as resprouting shrubs at ~ 2–4 m—we expect treefall rates of remaining large tree individuals to slow. Moreover, as bull elephants do most of the damage to trees (Stokke and du Toit 2000), information on age and size structure of the populations might have produced models with more explanatory power.

### Episodic versus Chronic Disturbances and Managing for Variability

One problem with using average elephant tree toppling rates to make these inferences is that elephants, as disturbances, are often highly stochastic in space and time: assuming a consistent annual tree turnover rate caused by elephants is unrealistic. As Figure S1 demonstrates, given enough time, ecosystems flattened by elephants, where impacts are higher than recovery rates, are likely also to experience periods of lower elephant impacts which would provide opportunities for recovery of the tree layer providing other browsers were not limiting recruitment (e.g. Pellew 1983). Managing the temporal aspect of elephant impacts is a huge challenge, especially in small reserves, but ultimately, as the numerous papers from the 1960s and 1970s eventually concluded, any land management plan aiming to accommodate elephants needs to accept that there will be times and places where elephant-caused tree losses are far higher than tree replacement rates (Figure S1). How to replicate these episodic and unpredictable disturbance regimes in ecosystems that currently lack elephants (i.e. how to fix the “no-elephant problem”) is a challenge for conservationists and land managers in Africa.

Humans have replaced elephants in most ecosystems. When we compare elephant-driven turnover rates to human fuel-wood harvesting, we find 2.61 million km^2^ of the region has human woodfuel use rates within the 25–75% bounds of what we estimate elephants would have been toppling in the past, i.e. woodfuel harvesting may be replacing an ecosystem process that is no longer present, and could be consistent with promoting ecological functioning, not a cause of degradation (Wells and others 2022). There is an equivalent area (2.38 million km^2^) where humans are removing far more biomass than elephants did on average, having impacts beyond what the ecosystem would have evolved with—likely to be unsustainable if they persist for too long. Moreover, an expansive 9.55 million km^2^ has human fuel-wood harvesting below what we predict elephants would have been removing, thus lacking an important driver of vegetation dynamics. In fact, landowners throughout Africa manage encroaching trees by reducing tree cover, sometimes by inviting local communities in to harvest the wood that is altering ecosystem functioning and ecosystem service provision (Birch and others 2016).

**Figure 3:**
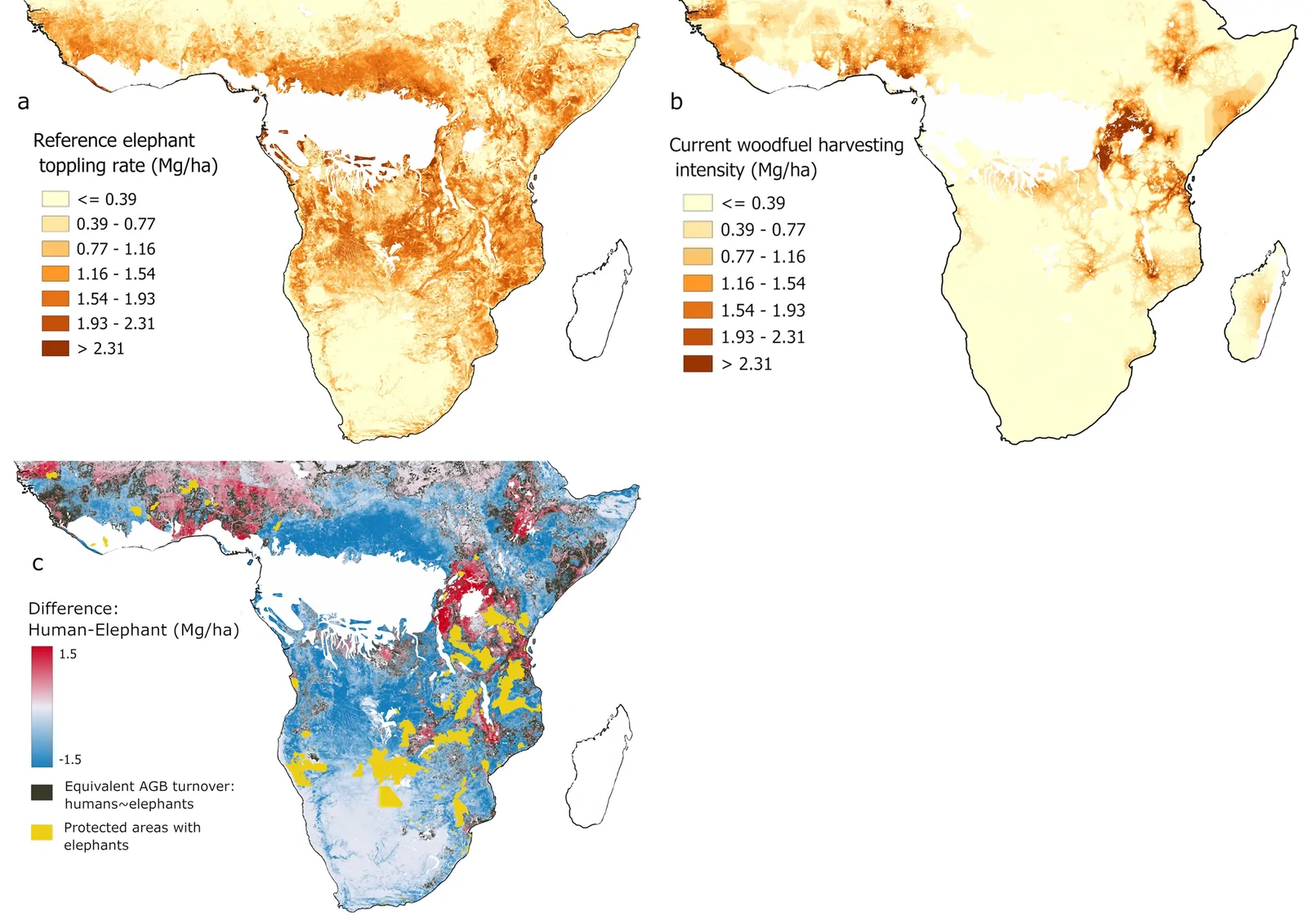
Above-ground biomass (AGB) loss due to **a** tree toppling by pre-colonial elephant populations, **b** current woodfuel harvesting by people, and c) difference between current human-caused AGB loss and pre-colonial elephant AGB loss. The landscapes where human and elephant impacts are equivalent are coloured in grey, and the landscapes where elephants still exist are coloured in yellow. Forest landscapes are excluded as we do not have data for elephant impacts in forest.

Of course, fully comparing human versus elephant impact on tree biomass requires that we also account for timber harvesting and other human-caused tree loss pathways (see methods). Moreover, while carbon cycling is important for understanding energy flows (Loft and others 2025; Pritchard and others 2018), it does not encompass important processes such as nutrient cycling and seed dispersal, where human and elephant impacts differ. By leaving the majority of the fallen stem in the landscape, elephants return carbon and nutrients to the ecosystem and create small-scale herbivore-exclusion zones where seedlings can recruit safe from the effects of other herbivores (Abraham and others 2023). Their actions in bark stripping and snapping of stems can alter tree structure to create habitats for cavity-using fauna, and they are known to be important dispersal agents for several species that humans do not use/disperse (Bunney and others 2019). Nevertheless, the biomass data presented here do force us to reassess common definitions of degradation, which is often defined as a reduction in above-ground biomass density (e.g. McNicol and others 2018). Too few disturbances can also reduce ecosystem functioning: Both the red and the blue areas in Figure 3C could be considered degraded, but for different reasons. The challenge in savannas and woodlands is to manage for a dynamic tree layer, enabling periods of intensive use and periods of recovery.

Most past analyses on elephant impacts have focused on landscape-scale patterns and demonstrate strong spatial heterogeneity (e.g. Mograbi and others 2017; du Toit and others 2014; Teren and others 2018). Elephant toppling rates increase close to water—parts of the landscape where regeneration is also limited due to high numbers of other herbivores. Like elephants, human impacts are also localised: they are concentrated near towns and roads rather than rivers, but can become widespread at higher human densities (Adrián Ghilardi and others 2016). This study explicitly averaged over local-scale variability and could not investigate species-specific impacts but these will be important to consider when assessing appropriate tree turnover dynamics in conservation and restoration projects (O’Connor 2017).

### Defining and Modelling Elephant-Tree Interactions

Our analysis highlighted a perplexing problem with modelling the impacts of elephants on trees: should this be represented as a constant rate, or relative to the number of trees in an ecosystem?

**Figure 4:**
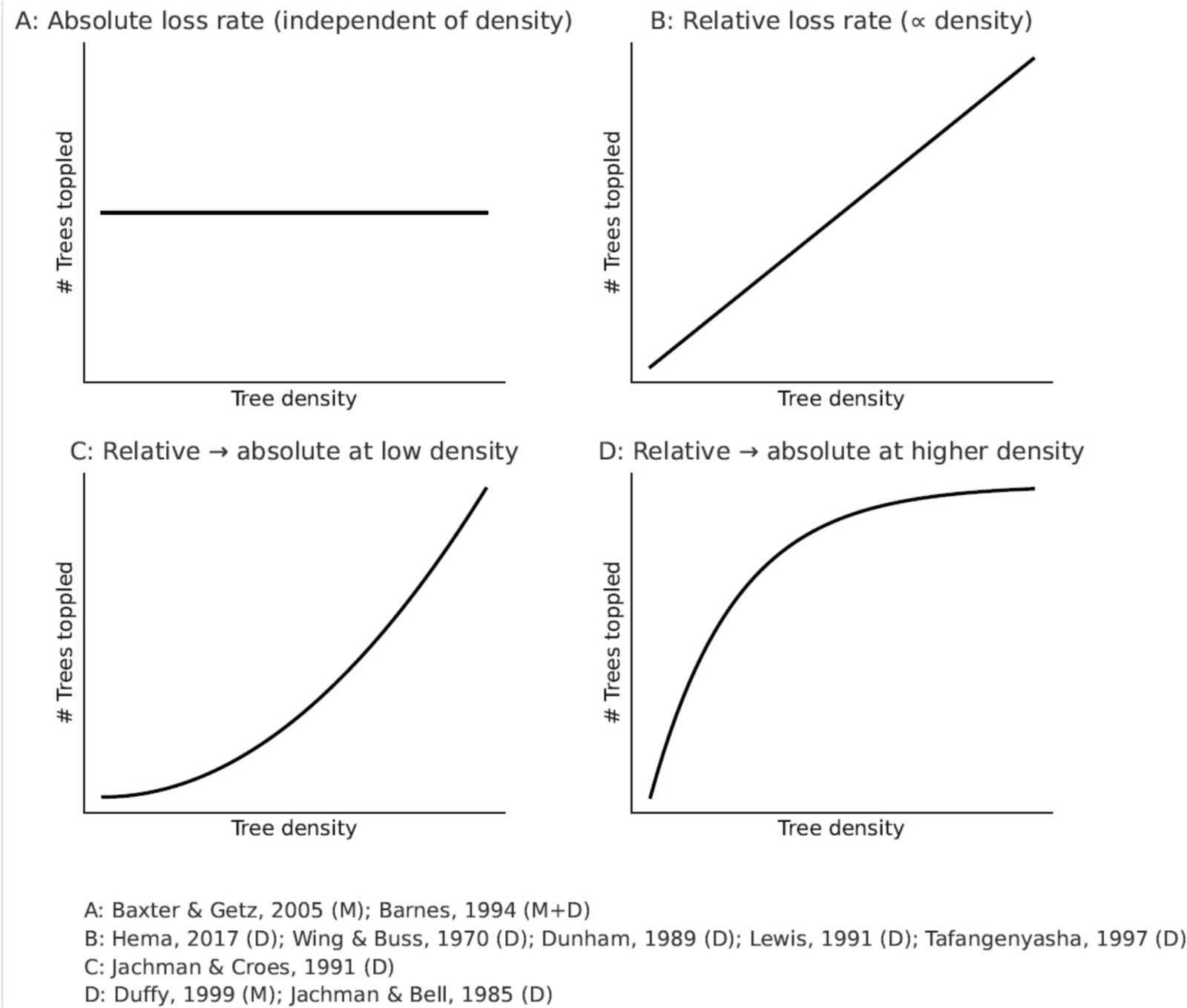
Four ways that elephant impact in response to tree density has been demonstrated and/or conceptualised in the literature so far. *M* = model, *D* = Data. All graphs assume a stable elephant population. Panel B has the most empirical support although this implies that elephants can never destroy the resource. Where elephants create browsing lawns and substitute use of large trees through utilisation of shrubs and coppice (Lewis 1991, O’Connor and others 2024), patterns such as shown in C might emerge. Theoretically, saturation at high tree densities (D) is likely, but further investigation is needed.

Loss rates that are relative to tree density (Figure 4B) imply that elephants can never entirely destroy the resource, as their usage decreases with availability (Barnes and others 1994). Moreover, if high treefall rates result in density-dependent negative effects on elephant populations, then some form of Lotka–Volterra dynamics are possible: the system could show lagged responses with periods of tree loss and recovery over very long time-scales. Alternatively, if elephant impacts were totally independent of tree cover (Figure 4A) then dramatic and potentially irreversible switches from high to low tree cover would be expected, and managers would need hands-on management of elephants to avoid these tree population crashes (Scholes and Kruger 2011).

The majority of the literature supported the response as shown in Figure 4B—that elephants knock down more trees when there are more trees available (Hema and others 2017; Lewis 1991; Tafangenyasha 1997; Wing and Buss 1970; Dunham 1989). However, there has been far more research into what elephants do in dwindling tree populations than in high tree cover ecosystems. It is unlikely that elephant use could expand linearly with the resource at very high tree density, so the saturating functional response modelled by Duffy and others (1999) is more likely, but it remains to be tested. In low tree cover systems, the response could depend on whether alternative forage is being provided (e.g. by shrubs and coppice shoots in the Lewis 1991 example) or not (e.g. for Baobabs in the Barnes (1994) example). Thus, it is likely that at both low and high tree cover the linear relationship is lost (i.e. some mixture of Figures 4C and D is probably more likely).

However, there is much room for both theoretical and empirical research to help resolve these questions, and this is an important area for further research, especially for predicting impacts among different ecosystems, rather than within ecosystems where most of the elephant research has so far been focused (Owen-Smith 2007).

It is possible that the elephant impacts in this dataset are inflated: we used relative, not absolute measures of tree loss for this calculation, so might be over-estimating impacts in high-biomass ecosystems if elephant toppling rates saturate at high tree densities (Omeja and others 2014). Moreover, reporting bias might have skewed our dataset towards situations where elephants are having higher than normal impacts on vegetation. Finally, as argued by many researchers in the 1960s, and shown by Guldemond & van Aarde (2008), elephant behaviour might be more extreme when they are confined to small areas and not able to move freely through landscapes.

Nevertheless, the toppling rates reported here are consistent with what is observed in ecosystems with elephants and align with independent datasets on tree turnover and NPP across tropical savannas and woodlands (Godlee in review). Moreover, as discussed above, historical accounts of elephant densities and impacts prior to the impact of ivory trading report similar elephant densities and impacts to what was reported in our meta-analyses. The dataset we used is small, and we encourage people to produce more data, from a wider range of landscapes, especially those where elephant densities are low, or elephant impacts are difficult to observe. This should include estimating elephant densities and impacts on long-term plots, such as the SEOSAW network (Partnership 2021). To fully test the hypothesis that impacts will vary depending on the proportion of trees available in preferred size classes, we should quantify elephant impacts in environments with different tree size distributions and expand the spatial scope of our observation data beyond the few highly studied landscapes available currently. These approaches will enable a more nuanced exploration of current and pre-colonial elephant impacts, which can be used to infer the desirable tree turnover rates for ecosystems not only in Africa, but globally, where these megaherbivores have been eliminated.

## Conclusion and Implications

Here, we employ crude yet quantitative methods to assess levels of human tree removal that are within the bounds of variation these ecosystems evolved with. It is known that elephants routinely act to switch ecosystems from one structural state to another, and our analysis identifies tree turnover rates that can sometimes exceed replacement rates. Yet, when human activities remove trees and switch states, this is usually termed degradation. There is growing recognition that people living in landscapes can help to maintain ecosystem functioning (Martin and others 2021; Reyers and Bennett 2025) and that our concept of “natural” is distorted by the loss of the global megafauna (Søndergaard and others 2025; Owen-Smith 1987). Quantitative data such as presented here can help to change perceptions and provide guidelines for managing multi-functional landscapes.

When landscapes are characterised as degraded it can encourage transformation to other land uses, or inappropriate restoration activities (Veldman 2016). Low-biomass coppicing shrublands prevail in many parts of Africa, particularly those with high human impact, and yet, their plant species composition remains intact (Gotore and others 2021). Rather than viewing these landscapes as available for conversion to other land uses, we should recognise their ecological and functional importance and find ways of enabling the spatial and temporal disturbance dynamics that once defined them. Elsewhere, woody thickening is driving a different forms of land degradation, often requiring manual intervention to maintain open savannas and woodlands (Buisson and others 2021). Support from conservation authorities and the international community would be most valuable for these efforts, which, as we demonstrate here, are both appropriate and necessary unless we can reintroduce elephants into all our landscapes.

Both elephant and human impacts on savannas therefore call into question the definitions often used to identify degradation and sustainable use, and there is much work to be done to provide more useful and appropriate guidance to land managers on this continent and elsewhere.

## Supplementary Information

Below is the link to the electronic supplementary material.

Supplementary file1 (XLSX 24 kb)

Supplementary file2 (DOCX 387 kb)

## Data Availability

Data for this study are contained in Supplemental Table S1.
