# Supplementary material for: The “Too-Few Elephant Problem”: Quantitative Analysis of Historical Elephant Tree-Toppling Rates Across Africa
Source: Ecosystems. 2026 Aug 20;29(5):79. doi: 10.1007/s10021-026-01089-5 (PMC13493457; doi:10.1007/s10021-026-01089-5)
Supplement: Supplementary file 1 [file 10021_2026_1089_MOESM2_ESM.docx]

Table S2: *Summarising how the biomass of a savanna landscape is distributed among the different size classes of stems utilised by elephants (from 426 plots across southern Africa: 62982 stems, 45418 trees), as well as the percentage of toppled trees from each size class (elephant size class preference from Mtsetfwa and Archibald* (2026)*.*

| **Size class height (m)** | **Size class DBH (cm)** | **Median (IQR) AGB per tree (Mg)** | **Observed proportion total AGB** | **Proportion toppled trees** |
| --- | --- | --- | --- | --- |
| 0-3m | 0-6 cm | 0.0028 (0.000- 0.005) | 0.01 | 0.20 |
| 3-9m | 9-40 cm | 0.121 (0.023 –0.137 ) | 0.64 | 0.65 |
| >9m | >40 cm | 1.818 (1.129 - 2.055) | 0.35 | 0.15 |

Table S3: Comparing the performance of different models to test for an effect of i) elephant presence, ii) elephant density, and iii) vegetation type on tree toppling rates. The model with elephant presence alone had the highest explanatory power.

| **Name** | **AIC** | **AIC_wt** | **AICc** | **AICc_wt** | **BIC** | **BIC_wt** | **R2** | **RMSE** | **Sigma** | **Model type** |
| --- | --- | --- | --- | --- | --- | --- | --- | --- | --- | --- |
| ElephantYN | -284 | 0.84 | -283 | 0.92 | -277 | 1 | 0.59 | 0.04 | 0.59 | Gamma (log link) |
| ElephantYN and Veg type | -281 | 0.16 | -279 | 0.08 | -265 | 0 | 0.62 | 0.04 | 0.60 | Gamma (log link) |
| Elephant Density | -242 | 0 | -242 | 0 | -236 | 0 | 0.12 | 0.04 | 0.74 | Gamma (log link) |
| Null | -238 | 0 | -238 | 0 | -233 | 0 | 0 | 0.04 | 0.73 | Gamma (log link) |
| Elephant Density and Veg type | -236 | 0 | -234 | 0 | -221 | 0 | 0.15 | 0.04 | 0.77 | Gamma (log link) |

*Figure S1. Comparing the biomass loss from elephants estimated in this paper with modelled woody AGB productivity from Godlee (in review). Orange line represents a loess smooth of the median woody biomass loss from elephants of 1000 random points across a rainfall gradient. Grey bars show the 25^th^ quantile and 75^th^ quantiles for the same points – capturing the large variation in observed elephant impacts. The green line shows the modelled NPP across tropical savannas and woodlands from Godlee et al (in review).* *On average woody productivity is higher than the elephant loss rates across all rainfall regimes, but elephant loss rates at any one point in space and time can often exceed replacement rates. Note, the orange line does not include background stem loss rates rates (estimated here as 0.62% of trees and by Godlee as ~1% of AGB).*

Figure S2: (Reproduced from Mtsetfwa and Archibald (2026)) Summary of meta-analysis on the how the probability of top-kill by six dominant top-kill agents changes along the life history of a savanna plant. Arrows indicate reported cut-off sizes for targeted tree size (DBH, cm).

*Figure S3: Where both metrics are available, it is possible to compare absolute and relative loss rates. Low relative loss rates always imply low absolute loss rates, but high relative loss rates can result in low or high absolute loss rates.*

*Figure S4. Comparing estimated background tree loss rates with landscapes where only elephant treefall was measured, and landscapes where all treefall was recorded (including both elephants and background rates of damage). The mean damage in landscapes with elephants is higher than the sum of the elephant damage and the background damage, implying that the background damage rates (caused by fire/termites/windthrow, etc) are also higher when there are elephants in the landscapes. This is supported by various papers showing interactive effects of elephants and fire, for example, but was not accounted for in this analysis, as it would require more rigorous testing.*

Figure S5: Showing a) how much of the above-ground biomass is contributed by trees of different size classes in savanna/woodland landscapes in Africa (quantified from SEOSAW data) and b) elephant preference for trees of different size classes (from Mtsetfwa and Archibald (2026)). These data were used to convert individual tree toppling rates into a measure of biomass turnover rates. See methods for details.

Table S2: Model result on the effect of elephant presence on tree loss rates

Table S3: Model results on the effect of elephant density on toppling rates

Table S4: Model results for an effect of vegetation type on tree loss rates.

**References:**

Mtsetfwa, F. and Archibald, S. (2026) “Spatiotemporal variation in top-kill agents relative to ontogenetic resprouting strategies in African savannas,” *BioScience*, 76(4), pp. 385–396. Available at: https://doi.org/10.1093/biosci/biag008.
